# Determination of the ion collection efficiency of the Razor Nano Chamber for ultra‐high dose‐rate electron beams

**DOI:** 10.1002/mp.15675

**Published:** 2022-05-20

**Authors:** Marco Cavallone, Patrik Gonçalves Jorge, Raphaël Moeckli, Claude Bailat, Alessandro Flacco, Yolanda Prezado, Rachel Delorme

**Affiliations:** ^1^ Institut Curie, PSL Research University Radiation Oncology Department Proton Therapy Centre Centre Universitaire 91898 Orsay France; ^2^ Laboratoire d'Optique Appliquée ENSTA Paris École Polytechnique CNRS‑UMR7639 Institut Polytechnique de Paris 91762 Palaiseau Cedex France; ^3^ Institute of Radiation Physics Lausanne University Hospital and Lausanne University Lausanne, Rue du Grand‐Pré 1 Switzerland; ^4^ Institut Curie Université PSL CNRS UMR3347 Inserm U1021 Signalisation Radiobiologie et Cancer 91400 Orsay France; ^5^ Université Paris‐Saclay CNRS UMR3347 Inserm U1021 Signalisation Radiobiologie et Cancer 91400 Orsay France; ^6^ University of Grenoble Alpes CNRS, Grenoble INP, LPSC‐IN2P3 38000 Grenoble France; ^7^ Imagerie et Modélisation en Neurobiologie et Cancérologie (IMNC) CNRS Univ Paris‐Sud Université Paris‐Saclay 91400 Orsay France

**Keywords:** dosimetry, FLASH radiotherapy, ion recombination, ionization chamber, ultra‐high dose‐rate

## Abstract

**Background:**

Ultra‐high dose‐rate (UHDR) irradiations (>40 Gy/s) have recently garnered interest in radiotherapy (RT) as they can trigger the so‐called “FLASH” effect, namely a higher tolerance of normal tissues in comparison with conventional dose rates when a sufficiently high dose is delivered to the tissue. To transfer this to clinical RT treatments, adapted methods and practical tools for online dosimetry need to be developed. Ionization chambers remain the gold standards in RT but the charge recombination effects may be very significant at such high dose rates, limiting the use of some of these dosimeters. The reduction of the sensitive volume size can be an interesting characteristic to reduce such effects.

**Purpose:**

In that context, we have investigated the charge collection behavior of the recent IBA Razor™ Nano Chamber (RNC) in UHDR pulses to evaluate its potential interest for FLASH RT.

**Methods:**

In order to quantify the RNC ion collection efficiency (ICE), simultaneous dose measurements were performed under UHDR electron beams with dose‐rate‐independent Gafchromic™ EBT3 films that were used as the dose reference. A dose‐per‐pulse range from 0.01 to 30 Gy was investigated, varying the source‐to‐surface distance, the pulse duration (1 and 3 μs investigated) and the LINAC gun grid tension as irradiation parameters. In addition, the RNC measurements were corrected from the inherent beam shot‐to‐shot variations using an independent current transformer. An empirical logistic model was used to fit the RNC collection efficiency measurements and the results were compared with the Advanced Markus plane parallel ion chamber.

**Results:**

The RNC ICE was found to decrease as the dose‐per‐pulse increases, starting from doses above 0.2 Gy/pulse and down to 40% of efficiency at 30 Gy/pulse. The RNC resulted in a higher ICE for a given dose‐per‐pulse in comparison with the Markus chamber, with a measured efficiency found higher than 85 and 55% for 1 and 10 Gy/pulse, respectively, whereas the Markus ICE was of 60 and 25% for the same doses. However, the RNC shows a higher sensitivity to the pulse duration than the Advanced Markus chamber, with a lower efficiency found at 1 μs than at 3 μs, suggesting that this chamber could be more sensitive to the dose rate within the pulse.

**Conclusions:**

The results confirmed that the small sensitive volume of the RNC ensures higher ICE compared with larger chambers. The RNC was thus found to be a promising online dosimetry tool for FLASH RT and we proposed an ion recombination model to correct its response up to extreme dose‐per‐pulses of 30 Gy.

## 1 INTRODUCTION

The use of ultra‐high dose‐rate (UHDR) irradiations (mean dose rate ∼ > 50 Gy/s) have gained recent interest in radiotherapy (RT) since they have been shown to induce the so‐called “FLASH‐effect,” namely a remarkable reduction of normal tissue complication probability compared with conventional dose rate (CONV) regimens (≤0.03 Gy/s), while preserving a comparable tumor response.^1^ The FLASH effect has been demonstrated in several animal studies of various types (fish eggs, mice, cats, mini‐pigs, etc.),^2^, ^3^, ^4^, ^5^, ^6^, ^7^ and a first patient (skin melanoma) has been treated recently.^8^, ^9^ Although some FLASH effects have been also observed with photon^10^ and proton^11^ beams, the most established demonstrations were obtained with low‐energy electron beams (<20 MeV), limiting their current application to superficial tumors. New developments in compact high‐gradient accelerator techniques^12^, ^13^, ^14^ and laser‐driven electron beam technologies^15^, ^16^, ^17^ allow to consider the use of very high‐energy electron (VHEE, *E* > 70 MeV) beams^18^, ^19^, ^20^ to address the penetration depth limitation and exploit the FLASH effect for the treatment of deep‐seated tumors. However, such beams typically present ultra‐short pulse durations (ns to fs) and potential dose rates within the pulse as high as 10^10^ Gy/s, which add dosimetry challenges to UHDR irradiations.^21^, ^22^ Proton pencil beam scanning can also be a promising solution to trigger a FLASH effect in deep‐seated tumors as the facilities already exist.^23^

The clinical transfer of FLASH RT^6^, ^24^ requires the development of accurate and practical dosimetry tools to ensure the safe delivery of the electron FLASH irradiation. Current dosimetry protocols^25^, ^26^ and codes of practice are not suitable for such high dose rates. Although passive dosimeters, such as radiochromic films, alanine, or thermoluminescent detectors, are currently being used for UHDR dosimetry,^8^, ^27^, ^28^ ion chambers remain the gold‐standard detectors in RT. They offer metrological traceability and practical clinical use as they do not require postirradiation processing. However, commonly employed ion chambers show non‐negligible ion recombination when the dose‐per‐pulse (DPP) increases far beyond conventional RT dose rates.^29^, ^30^, ^31^ This imposes the use of ion recombination models for UHDR irradiations, increasing the dosimetry uncertainty as well as accuracy. Some models have been proposed for plane parallel ion chambers as the PTW Advanced Markus^32^ or the ROOS^30^ chambers in UHDR electron beams.

The reduction of the sensitive volume of the ion chambers would reduce ion recombination at high DPP. Along this line, the new microchamber Razor™ Nano Chamber (RNC)^33^ by IBA (ion beam applications), having the smallest available active volume (3 mm^3^) among commercially available ion chambers, could be an optimum solution for online dosimetry of UHDR irradiations. Indeed, ion collection efficiency (ICE) and polarity effects of the RNC have already been characterized in CONV RT photon beams in flattening filter free irradiation modality, presenting higher mean dose rates (up to approximately 0.4 Gy/s) than CONV photon irradiation modality and reaching DPP up to 2.2 mGy.^34^, ^35^

The purpose of the present study was to characterize the response of the RNC in UHDR electron beams and to determine the ICE factors for DPP ranging from approximately 0.01 to 30 Gy.

## 2 MATERIALS AND METHODS

### 2.1 Dosimetry tools and irradiation facilities

The RNC prototype^33^ is an air‐filled microchamber presenting the same external geometry than the Razor Chamber cc01 (IBA Dosimetry, Schwarzenbruck, Germany), but with an active volume of 3 mm^3^ instead of 10 mm^3^, encapsulated between two spherical electrodes. The diameter of the active volume is 2 mm. The extremely small sensitive volume of the RNC enables its use for dosimetry in both photon and electron beams. In the present study, all measurements were performed using the same individual RNC (serial number 16232), with the recommended nominal polarizing voltage of +300 V. The chamber's signal was collected using a Dose 1 electrometer (IBA Dosimetry; serial number 26991) connected to the RNC by a shielded triaxial cable.

The chamber plus electrometer measurement chain was firstly characterized in CONV regimen with an 8 MeV electron beam using a clinical linear accelerator (LINAC) Elekta Synergy® (Elekta AB, Stockholm, Sweden).

The chamber's response was then characterized in UHDR regimen on the Oriatron eRT6 (PMB‐Alcen, France) experimental accelerator. The eRT6 is a prototype LINAC capable of delivering 0.5–4 μs electron bunches with DPP ranging from 10^−4^ to 100 Gy. The output beam intensity is tuned by independently setting the LINAC parameters, that is, the number of electron pulses (N), the repetition rate (*f*), the pulse duration (*w*), and the grid tension (GT). Both *w* and GT allow to regulate the accelerated charge of the beam pulse. Different combinations of these four parameters with the measurement source to surface distance (SSD) allow for the variation of a mean dose rate between 10^−2^ Gy/s (conventional mode) and 10^3^ Gy/s (UHDR mode). Additional information could be found in Jaccard et al.^36^

At UHDR, simultaneous measurements were performed with EBT3 radiochromic films (Gafchromic™ EBT3; Ashland Inc., Covington, KY, USA), which have been shown to be dose‐rate independent.^28^, ^32^, ^37^ After a calibration in CONV regimen, the films were used as the reference dose in UHDR regimen as there is currently no dosimetric metrological instrument traceable to a standard for such dose rates. The film handling and calibration procedure is described in detail in the next paragraph. The monitoring of the beam was performed with a beam‐current transformer (BCT)^38^ placed at the exit of the eRT6. The transformer's signal is proportional to the beam output at the exit of the LINAC and, consequently, to the delivered absorbed dose to water. The charge to dose calibration factor depends on the beam parameters, such as pulse duration or grid tension, and irradiation geometry such as collimator size or target distance.^39^ This limits the flexibility of the irradiation conditions.

### 2.2 Gafchromic film calibration

EBT3 film response was calibrated in absorbed dose to water in CONV mode with a 4 MeV electron beam produced by an Elekta Synergy® LINAC. Information about the procedure can be found in Jaccard et al.^28^ Briefly, for each batch, films were irradiated in a solid water phantom (RW3 slabs, PTW) in reference dosimetry conditions for this energy, that is, at 0.7 cm depth, using an SSD of 100 cm and a field size of 20 × 20 cm^2^. The films used for calibration have comparable sizes as well as exact same location on the scanner, in order to limit the errors coming from the inhomogeneity of the scanner. Ten dose points from 0.1 to 30 Gy were taken, with two films per dose point. The mean value between the two films was used for the calibration, with a standard deviation (SD) of less than 0.5% ($\sigma_{\mathrm{mean}\;\mathrm{film}\;\mathrm{calib}}$) between the two measurements. The reference dose is given by a parallel‐plate ionization chamber (NACP‐02; IBA Dosimetry GmbH), calibrated at the Swiss Federal Institute of Metrology (METAS) and traceable to international standards. A water‐to‐RW3 correction factor of 0.985 was applied, in order to use RW3 instead of water. We did not use the double exposure method to mitigate film homogeneity induced errors, because we took it into account during the uncertainty evaluation under repeatability testing. The uncertainty $\sigma_{\mathrm{Dose}}^{\mathrm{IC}}$ associated to the dose determination at the calibration time was evaluated to be 1.5%.

The films were scanned with an Epson Expression 10000 XL flatbed scanner (Epson, USA) in transmission mode, with a resolution of 300 dpi in 48 bits colors. The red channel was used because it provides the better sensitivity and lower uncertainty for low doses up to 10–14 Gy,^37^, ^40^, ^41^ which cover the major part of our measurements. The mean pixel value was extracted in a centered circular region of interest (ROI) of 2 mm of diameter, in order to have the same size than the RNC‐sensitive area. Irradiations were homogeneous over the film surface, leading to a maximum SD on the 2 mm ROI pixel values of 3% for doses above 0.5 Gy. The latter uncertainty σ_Film, SD_ combined with $\sigma_{\mathrm{mean}\;\mathrm{film}\;\mathrm{calib}}$ and $\sigma_{\mathrm{Dose}}^{\mathrm{IC}}$ as the quadratic sum led to a global uncertainty on dose,$\;\sigma_{\mathrm{Film}\;\mathrm{Dose}}$, of 3.4% for doses below 15 Gy. The calibration curve was determined with a 5‐degree polynomial fit of the dose delivered expressed as a function of the film optical density. Although the recommended dynamic range of EBT3 films is between 100 mGy and 15 Gy, we had to extend the calibration curve up to doses of 30 Gy in order to characterize the RNC at typical DPP used in FLASH therapy. To verify the reliability of the dose given by the red channel above 15 Gy, we have compared the given doses by those obtained with the green and the blue channel calibration curves, that have a higher sensitivity at high dose values.^40^ We noticed a maximum dose deviation, $\sigma_{\mathrm{Dose}}^{\mathrm{Ch}}$, of 5% with the red channel, which is compatible with previous studies by Jaccard et al.^28^ Hence, the quadratic sum of $\sigma_{\mathrm{Dose}}^{\mathrm{Ch}}$ and σ_Dose_ of 6% was used as the global uncertainty on doses from 15 to 30 Gy, $\sigma_{\mathrm{Film}\;\mathrm{Dose}>15\mathrm{Gy}}$.

### 2.3 Linearity and repeatability of the RNC response

#### 2.3.1 Measurements in CONV mode

Linearity and repeatability of dose measurements were performed at the Elekta Synergy® LINAC to verify the linear dependency with dose of the RNC charge collection and quantify the uncertainty of the measurement chain at a conventional dose rate. In addition, the linearity measurements allowed for determining the calibration coefficient of the RNC for low energy electrons $N_{\mathrm{RW}3,\;\mathrm{el}\;8\mathrm{MeV}}$, useful for comparison purpose with high dose‐rate behavior.

Linearity measurements were performed under reference calibration conditions for the 8 MeV electron beam of the LINAC. The RNC was placed in RW3 plates at a 1.7 cm depth, with an SSD of 100 cm and within a field size of 20 × 20 cm^2^. The mean dose rate was approximately 6 Gy/min. The dose delivered to the RNC was increased from 1 to 10 Gy. The given dose is traceable to international standards using a parallel‐plate ionization chamber (NACP‐02; IBA Dosimetry GmbH) calibrated at the METAS, and a water‐to‐RW3 correction factor of 0.985 was applied. Two RNC measurements per dose point were taken and the mean value was considered.

Although it is not fully applicable, we have followed the IAEA TRS‐398^36^ protocol methodology to determine the RNC dose response in RW3 material and in CONV regimen as:

(1) $$D_{\mathrm{RNC}}=M\cdot N_{\mathrm{RW}3,\mathrm{el}8\mathrm{MeV}}\cdot k_{T,P}\cdot k_{h}\cdot k_{\mathrm{elec}}\cdot k_{\mathrm{pol}}\cdot k_{s}$$

where *D*_RNC_ is the absorbed dose in Gy measured by the RNC, *M* is the collected charge in nC, $N_{\mathrm{RW}3,\;\mathrm{el}\;8\mathrm{MeV}}\;$is the calibration factor in Gy/nC measured in a solid water (RW3) phantom in an electron beam of 8 MeV, *k*_T, P_ is the temperature and pressure correction factor, *k*_h_ is the humidity correction factor, *k*_elec_ is the electrometer correction factor, *k*_pol_ is the polarity correction factor, and *k*_s_ is the ion recombination correction factor. The factors *k*_h_, $k_{\mathrm{elec}},\;k_{\mathrm{pol}}$ and *k*_s_ were assumed to be equal to 1, which lead to the following expression of the calibration coefficient:

(2) $$\begin{array}{c} N_{\mathrm{RW}3,\mathrm{el}\;8\mathrm{MeV}}=\frac{D_{\mathrm{RNC}}}{M\cdot k_{T,P}} \end{array}$$

To evaluate the uncertainty of the RNC and electrometer measurement chain, 10 independent measurements of a constant dose were performed in two series: one at a low dose of 50 Monitor Unit (MU), corresponding to approximately 0.5 Gy per measurement, and one at a high dose of 500 MU, corresponding to approximately 5 Gy per measurement. The set‐up was the same as for the linearity measurements.

#### 2.3.2 Measurements in UHDR mode

RNC linearity and repeatability of dose measurements in UHDR mode were performed at the Oriatron eRT6. We aimed at quantifying the uncertainty of the measurement chain in UHDR mode and at verifying the dose linearity of the RNC at UHDR, which implies that the pulses could be considered as independent (i.e., the interval between two pulses is longer than the ion collection time, typically of few microseconds, plus the time required by the electrometer chain to elaborate the signal) at the pulse frequency employed in the experiment (10 Hz). If this condition is met, recombination can be described as a function of the pulse parameters such as dose‐per‐pulse, dose rate within the pulse and pulse duration.^42^, ^43^

For linearity measurements, the RNC was placed in an RW3 phantom at a depth of 1.5 cm, corresponding to the maximum of the depth‐dose profile of the 6 MeV Oriatron electron beam, with an SSD of 0.5 m. Irradiations were performed by increasing the number of pulses from 1 to 128 using a GT of 200 V. Two series were performed with a pulse duration of 1 and 3 μs, providing DPP of 1.9 and 5.5 Gy, respectively, according to film measurements.

For the repeatability measurements, the RNC was placed at an SSD of 0.2 m. The chamber was irradiated with a single pulse per measurement, using a pulse duration of 3 μs and a GT of 120 V, which corresponds to a DPP of 3.8 Gy according to film measurements. Ten independent RNC charge measurements were taken with a simultaneous beam current measurement obtained with the current transformer to account for the inherent machine short‐term variability.

### 2.4 ICE determination through film and RNC simultaneous measurements in UHDR mode

**FIGURE 1:**
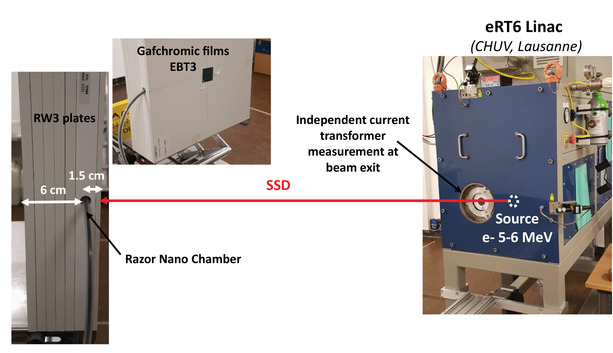
Experimental irradiation setup. Right: picture of the eRT6 Oriatron. Left: picture of the RW3 water equivalent phantom in which the Razor Nano Chamber was inserted. The picture also shows the EBT3 film placed at the phantom surface for simultaneous reference dose measurement. Series of measurements were performed with SSDs of 2, 0.5, 0.3, and 0.2 m

The RNC ICE in UHDR mode was determined at the eRT6 Oriatron using simultaneous film and chamber measurements performed in two independent experimental campaigns. The experimental set‐up is presented in Figure 1. The RNC was inserted in a tailored RW3 (water equivalent) phantom at 1.5 cm depth. Measurements were taken at four different SSDs, that is, 0.2, 0.3, 0.5, and 2 m, with two pulse durations of 1 and 3 μs, and varying the grid tension from 120 to 300 V in order to cover a DPP range between 10 mGy and 30 Gy. At each measurement point, that is, one combination of SSD, GT, and *w*, three measurements were performed at the nominal chamber polarization voltage of 300 V and corrected by the *k*_T, P_. For each measurement, the beam current was simultaneously measured with the BCT. One of the three RNC measurements per condition was completed with a simultaneous Gafchromic film (EBT3) measurement, placed at the surface of the RW3 phantom, which served as reference dose measurement. The two other RNC measurements performed without the film were corrected using the current transformer measurements. The pulse frequency was 10 Hz. The number of delivered pulses were varied from 1 at the shortest SSD to 100 at the largest SSD to keep the total dose within the dose range used for the EBT3 film batch calibration.

The DPP measured at the position of the RNC (1.5 cm of depth in the RW3 phantom) was determined from the film dose measured at the surface of the RW3, corrected by the ratio of the film dose at 1.5 cm to those at surface, *k*_F_. This specific correction factor *k*_F_ was determined at all SSD, GT, and *w* irradiation combination used, to account for the change in percentage depth dose (PDD) profiles. Indeed, as the SSD increase induces an increase of the beam size and a hardening of the energy spectra, the change of grid tension and pulse duration also affect the beam quality, as shown in Jaccard et al.^36^ Two independents *k*_F_ measurements were performed per (SSD, GT, w) condition, the mean of the two values for a given *k*_F_ was chosen and we retained the average SD, 2.5%, as the uncertainty on *k*_F_. The obtained table of *k*_F_ allowed to correct the DPP actually received by the RNC at each ICE measurement point.

The ICE was determined separately for the two pulse durations. It was calculated as:

(3) $$\begin{array}{ccc} \mathrm{ICE}\left(\mathrm{DPP}\right) & = & \frac{Q}{Q_{\mathrm{sat}}}\;\left(\mathrm{DPP}\right)\\ & = & \frac{Q\;\left[\mathrm{nC}\right]}{\frac{1}{N_{\mathrm{RW}3,\;\mathrm{el}\;\mathrm{eRT}6}\;\left[\frac{\mathrm{Gy}}{\mathrm{nC}}\right]\cdot k_{T,P}}\cdot \mathrm{DPP}\;\left[\mathrm{Gy}\right]} \end{array}$$

where *Q* is the charge collected by the RNC and *Q*_sat_ is the saturation charge, that is, the charge that would be collected to the chamber electrodes if no recombination occurs. *Q*_sat_ is determined by fitting the collected charge as a function of the DPP with a linear equation, at the largest SSD of 2 m, where recombination can be considered negligible (*Q* < 20 pC^32^). The collected charge was corrected for the day‐to‐day temperature and pressure variation. No polarization factor was computed and used to correct the expression on the right side of Equation 3 because of the difficulty, on such experimental UHDR facility, to decorrelate the actual recombination charge effect to potential polarization bias under chamber saturation condition without adding higher uncertainties. The ICE can be therefore considered as the contribution of both recombination and polarization to the total chamber saturation, as follows:

(4) $$\begin{array}{c} \mathrm{ICE}\left(\mathrm{DPP}\right)=\frac{1}{k_{s}\cdot k_{\mathrm{pol}}} \end{array}$$

Although the ICE is usually referred as the inverse of the recombination coefficient *k*_s_, we employed this term to indicate that the results still represent a deficit of collected charge. A detailed discussion on this choice can be found in section 4 .

The resulting ICE experimental points were fitted using the empiric logistic model proposed by Petersson et al.^32^ for the Advanced Markus chamber, also employed in other studies (e.g., McManus et al.^30^), whose equation is:

(5) $$\begin{array}{c} \mathrm{ICE}\left(\mathrm{DPP}\right)={\left(1+{\left(\frac{\mathrm{DPP}}{\gamma }\right)}^{\alpha }\right)}^{\beta } \end{array}$$

where α,β, and γ are fitting parameters with no explicit physical meaning.

This logistic recombination model has been shown in previous studies^30^, ^32^ to fit better the results of ICE for UHDR as compared to other models as those proposed by Boag et al.^44^, Burns & McEwen^45^ or Di Martino et al.^46^ In contrast to such theoretical models, the logistic model does not involve the bias voltage of the chamber in the formula and we have thus calculated the ICE only at the RNC recommended bias voltage of 300 V in view of its practical use in FLASH regimen.

### 2.5 Determination of uncertainties on dose and ICE measurements

Some uncertainties, presented as standard uncertainty (k=1) if not stated otherwise, affect the measurement of the dose delivered and of the RNC collected charge, finally all contributing to the uncertainty on the ICE determination. These uncertainties are discussed below and summarized in Table 1.

**TABLE 1:** Uncertainties on various parameters finally affecting the ICE uncertainty

Measurements affected by uncertainties	Contributions	Description	Uncertainty	Total uncertainty (method)
EBT3 dose	$\sigma_{\mathrm{mean}\;\mathrm{film}\;\mathrm{calib}}$	SD between two films during calibration	0.5%	0–15 Gy: 3.4% ; 15–30 Gy: 6% (Quadratic sum)
	$\sigma_{\mathrm{Dose}}^{\mathrm{IC}}$	Uncertainty on IC dose during calibration	1.5%	
	σ_Film, SD_	Inhomogeneity of films during UHDR irradiations (SD in 2 mm diameter)	3%	
	$\sigma_{\mathrm{Dose}}^{\mathrm{Ch}}$	Deviation between channels above 15 Gy	5%	
Dose at RNC position	$\sigma_{\mathrm{EBT}3\;\mathrm{dose}}$		3.4% (0–15 Gy) ; 6% (15–30 Gy)	0–15 Gy: 4.2% ; 15–30 Gy: 6.5% (Quadratic sum)
	σ_kf_	Depth dose corrections—SD between two measurements	2.5%	
*Q*_sat_	σ_Q_	Repeatability of RNC response	0.4%	2.5% (Monte Carlo approach)
	$\sigma_{\mathrm{Dose},\;\mathrm{RNC}\;\mathrm{position}}$		4.2% (0–15 Gy) ; 6.5% (15–30 Gy)	
ICE	σ_Q_	Repeatability of RNC response	0.4%	5.8% (*k* = 2) (sum)
	σ_Qsat_		2.5%	
ICE fitting curves	σ_ICE_		5.8%	1–8% (Monte Carlo approach)
	$\sigma_{\mathrm{Dose},\;\mathrm{RNC}\;\mathrm{position}}$		4.2% (0–15 Gy) ; 6.5% (15–30 Gy)	

Sources of error contributing to the global uncertainty on the dose delivered at the position of the RNC are the uncertainty related to the film response, estimated to be 3.4% below 15 Gy and 6% between 15 and 30 Gy (see section 2.2), and the uncertainty on the *k*_F_ factor used to correct for the PDD, estimated as the SD between the two measurements performed at each condition, and found to be of 2.5%. A cumulative error of 4.2 and 6.5% on the dose delivered at the RNC position was obtained for doses below and above 15 Gy, respectively.

The uncertainty on the ICE measurements is given by the sum of the uncertainties on the collected charge *Q* and on *Q*_sat_. The uncertainty on *Q* was estimated using the repeatability measurements and found to be 0.4% (see section 3.2). The percent error on *Q*_sat_ has been evaluated through a Monte Carlo approach according to GUM recommendation^47^: around each of the experimental points taken at the SSD of 2 m used for the linear fit determining *Q*_sat_, 5000 random points were generated using a 2D Gaussian distribution, with horizontal and vertical σ corresponding to the uncertainties on dose (4.2%) and on the RNC collected charge (0.4%) respectively. For each of the 5000 series of points, a linear fit was calculated and the SD of the 5000 values of angular coefficients was retrieved. A value of 2.5%, was obtained and used to quantify the uncertainty on *Q*_sat_. Altogether, the total uncertainty on the ICE can be estimated at 2.9% and a conservative value of 5.8% (k=2) was used.

We evaluated with a similar approach the uncertainty on the logistic curves used to fit the ICE experimental points as a function of the DPP. 1000 random points were generated around the ICE experimental values using a 2D Gaussian distribution having as horizontal and vertical σ the uncertainties on dose (4.2 and 6.5% below and above 15 Gy, respectively) and ICE (5.8%), respectively. Each series of points was fitted with a logistic model, resulting in 1000 fitting curves, that is, 1000 values of ICE for a given dose point. The SD between the ICE values for each dose point, ranging from about 1% at low doses and 8% at high doses, is used to quantify the uncertainty on the ICE curves presented in section 3.3.

## 3 RESULTS

### 3.1 RNC response at low dose rate

**FIGURE 2:**
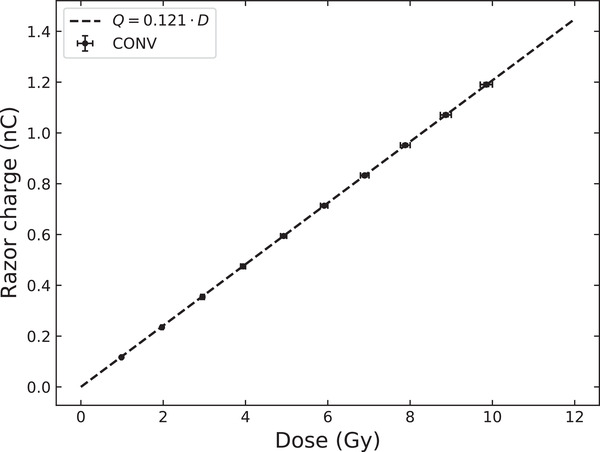
Linearity measurement to calibrate the RNC response under reference electron beam of 8 MeV delivered at conventional dose rate (Elekta Synergy, CHUV). The uncertainty on the dose was of 1.5% (horizontal error bars), and that on the RNC charge response was of 0.6% (vertical error bars)

Figure 2 presents the response of the RNC as a function of the dose in CONV mode. The RNC response is linear (*R* ^2^ = 1) in CONV mode within the dose range of 1–10 Gy. From this measurement, the calibration coefficient $N_{\mathrm{RW}3,\;\mathrm{el}\;8\mathrm{MeV}}$ of the RNC for an electron beam of 8 MeV was determined according to Equation 2, and found to be equal to 8.285 Gy/nC.

The repeatability measurements showed an SD of 0.6% (resp. 0.08%) for doses of about 0.5 Gy (resp. 5 Gy). We retained 0.6% for the uncertainty on the RNC charge measurement in CONV regimen.

### 3.2 RNC response in UHDR mode

**FIGURE 3:**
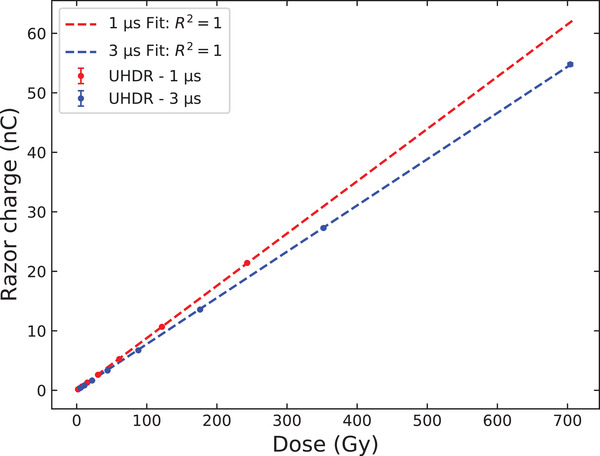
Linearity: Charge RNC response as a function of the total dose delivered at high dose rate with the eRT6 accelerator. The beam parameter used were a frequency of 10 Hz, a Grid Tension of 200 V and an SSD of 0.5 m. Two series were performed at a pulse duration of 1 and 3 μs, providing DPP of 1.9 and 5.5 Gy, respectively. The uncertainty on the RNC charge response was of 0.43% (vertical error bars)

Figure 3 presents the linearity measurement result of the RNC response as a function of the cumulated dose delivered in UHDR mode. The cumulated dose is increased by increasing the number of pulses without changing the beam parameters, that have been chosen with a frequency of 10 Hz, a grid tension of 200 V and an SSD of 0.5 m. The responses of the RNC and electrometer measurement chain are linear for both pulse durations. This result establishes dose linearity under UHDR conditions and shows that the pulses can be considered as independent at the chosen frequency.

Concerning the repeatability measurements performed in UHDR mode (for a DPP of 3.8 Gy), an SD of 1.0% was obtained. After correcting for the beam output variation using the BCT, the relative SD was reduced to 0.4% which we attributed to the RNC charge measurement uncertainty at UHDR.

**FIGURE 4:**
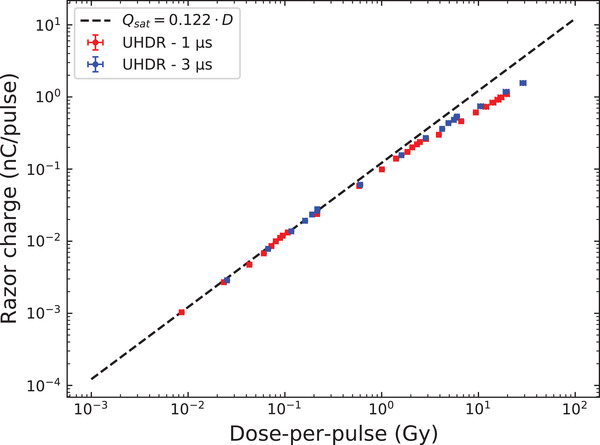
RNC charge response as a function of the dose‐per‐pulse (DPP). Two series were performed at pulse lengths of 1 μs (red dots) and 3 μs (blue dots), with DPP ranging from 0.01 to 30 Gy. The black linear dashed curve represents the saturation charge $Q_{\mathrm{sat}}$, that is, the charge that would be collected to the RNC electrodes if no recombination occurred. The uncertainty on DPP was of 5% (horizontal error bars) and that on the RNC charge response was of 0.43% (vertical error bars)

The result of the RNC charge response (*Q*) as a function of the DPP (DPP = 0.01 to 30 Gy) is reported in Figure 4. One can observe that the RNC data points follow the saturation charge *Q*_sat_ without recombination up to DPP of approximately 200 mGy, and start to differ increasingly from the linear tendency of *Q*_sat_ for higher doses, highlighting the occurrence of charge recombination inside the RNC sensitive cavity. Fitting linearly the data points at the SSD of 2 m, a calibration coefficient $N_{\mathrm{RW}3,\;\mathrm{el}\;\mathrm{eRT}6}$ was determined as the inverse of the angular coefficient of the fit (see section 2.4) and was found to be equal to 8.197 Gy/nC. This result can be compared with the calibration coefficient obtained under the 8 MeV conventional electron beam of the Elekta Synergy LINAC (8.285 Gy/nC), if we assume a *k*_pol_ = 1. The coherence between these two factors indicate the robustness of the film dosimetry used at the ert6.

### 3.3 ICE determination of the RNC

**FIGURE 5:**
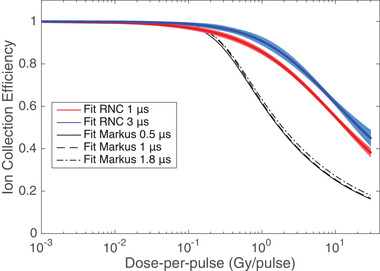
Ion collection efficiency of the RNC as a function of the dose per pulse, for pulse lengths of 1 μs (red curve) and 3 μs (blue curve), fitted with the logistic recombination model. The uncertainties on ICE (see section 2.5) are represented by the shaded region around the main curves. The RNC ICE results are compared with those of the Markus Advanced ion chamber of PTW for pulse lengths of 0.5–1.8 μs, as published by Petersson et al.^32^

The results of the RNC response as a function of the DPP shown in Figure 4 were used to obtain the ICE according to Equation 3. The resulting curves for the 1 and 3 μs pulse durations were fitted with the logistic model (Equation 5), whose fitting parameters are listed in Table 2 with their respective coefficients of determination *R* ^2^. The Figure 5 shows the ICE curves for the 1 μs (red curve) and 3 μs (blue curve) pulse durations compared to the ICE of the larger Advanced Markus Chamber taken from the previous work of Petersson et al.^32^ The RNC curves lie above the Advanced Markus chamber curves, which indicates that the small RNC volume ensures a lower recombination and can measure accurately higher DPP. For example, at 1 and 10 Gy per pulse, the measured ICE was found around 60 and 25%, respectively, for the Markus chamber, while still higher than 85 and 55%, respectively, for the RNC with both pulse durations. However, the RNC seems more sensitive to the pulse duration in view of the larger separation between the curves of different pulse duration compared with the Advanced Markus chamber for which the curves are almost identical. We observed for the RNC that the ICE for a given DPP decreases with the pulse duration reduction.

**TABLE 2:** Parameters of the fit obtained for 1 and 3 μs with the logistic recombination model proposed by Petersson et al.^32^ and described in Equation 5

Parameters	*α*	*β*	*γ*	*R* ^2^
Fit 1 μs	0.76	0.61	5.12	0.97
Fit 3 μs	0.97	0.35	3.28	0.94

## 4 DISCUSSION

The development of adapted methods and practical tools for online UHDR irradiation dosimetry is a current challenge for FLASH RT clinical transfer.^22^ In that context, we have investigated the potential interest of using the new RNC for FLASH RT. Since the RNC has been recently developed, we first characterized its response with a low dose rate electron beam produced by a conventional LINAC. In particular, we verified that a correct linear response was obtained with increasing dose and that the reproducibility of the charge response to repetitive independent measurements showed less than 1% deviation, as is expected by clinical tools. The linearity and repeatability results obtained at the Elekta LINAC were found coherent with those obtained at the experimental Oriatron LINAC, and confirmed the robustness of the dosimetry protocol used for the chamber characterization performed at high dose rate. The ICE obtained for the RNC was shown to be superior in comparison with that of the advanced Markus chamber, for dose‐per‐pulses ranging from 0.2 to 30 Gy. This result confirms the hypothesis that a smaller sensitive volume reduces recombination, which can be explained by considering the shorter time that the secondary charges spent to reach the electrodes. In addition, as stated previously, this volume reduction did not impact the performance of the RNC at conventional dose rates. Another advantage of the RNC over the Markus chamber is that the ICE slope is flatter at high DPP, which decreases the dose correction uncertainty.

These ICE results have been obtained without decorrelating the polarization factor $k_{\mathrm{pol}}\;$from the recombination factor *k*_s_. This choice was motivated by the fact that separately measuring the *k*_pol_ would be at the price of adding larger uncertainties on the overall correction to the chamber response. First, there might be a potential dependency of the saturation effect with the polarity which would be not trivial to decorrelate. Second, several independent parameters have to be tuned on the eRT6 facility to cover the whole range of dose per pulses required, and this might induce some slight beam variations (in terms of beam shape, spectra, pulse intensity, etc.) that could also affect the polarity. Therefore, although it would be beneficial to measure such a polarity factor independently according to the dose‐per‐pulse on a clinical facility dedicated to FLASH with minimum tunable parameters, we believe that separating the two effects in the present study would be counterproductive for a practical characterization of this chamber in unconventional regime as it could depend on the very specific parameters used on the eRT6 and would add larger uncertainties. We have thus chosen to measure a charge collection that might be due to both phenomena rather than recombination alone. However, according to Petersson et al.,^32^ maximum polarization factors from 5 to 9% were measured with the Advanced Markus chamber, which correspond to ICE of 0.2–0.3. This suggests that chamber saturation is widely due to an increase of the recombination process rather than a polarity bias and, based on this evidence, it can be reasonably assumed that the polarity effects would affect similarly the RNC charge collection, or even less as the microchamber saturate less than the Markus at same DPP.

Besides, our results also indicate that in this pulsed temporal operation mode, that is, 1–3 microsecond pulse duration range, the small sensitive volume of the RNC suffers from a higher sensitivity to the pulse duration. Indeed, ICE for a given DPP decreased with the pulse duration reduction, that is, with the increase of the dose rate within the pulse. On the contrary, the Advanced Markus chamber curves for different pulse durations are only slightly separated, which indicates that ion recombination in the Markus chamber's active volume is less sensitive to the dose rate within the pulse. This behaviour may be explained by comparing the chamber ion collection time to the pulse duration. If the latter is much shorter than the ion collection time, the charge production in the chamber sensitive volume can be considered instantaneous regardless of the pulse duration. As a result, recombination should exclusively depend on the total dose delivered within the pulse.^42^ The Advanced Markus has a space of 1 mm between its electrodes and an ion collection time around 20 μs,^48^ whereas the RNC, whose electrode distance is two times smaller, has an ion collection time of approximately 5 μs. Therefore, a microsecond pulse can be considered almost instantaneous if compared with the Advanced Markus collection time whereas it is comparable to the RNC collection time. This effect might explain the highest RNC sensitivity to the pulse duration. As a consequence, a careful characterization of the recombination correction factor for different pulse durations would be mandatory for such a chamber in order to transfer its use for dosimetry of beams featuring shorter pulses (in the ns to fs range), such as laser‐driven beams^15^, ^21^, ^49^ or other VHEE accelerators.^12^, ^13^, ^14^ Further investigations will be dedicated to determine below which pulse duration the RNC, as well as other small chambers, become insensitive to the dose rate within the pulse. In this case, the chamber calibration against the DPP for a given pulse duration could be directly transferred to shorter pulse durations. For instance, the study of Gotz et al.^48^ demonstrated that the response of the Advanced Markus Chamber becomes insensitive to the dose rate below a pulse duration of around 1 microsecond. We can expect from our result that such a limit would be even below 1 μs for the RNC. This could constitute an important prospect of the present study, as VHEE beams have been receiving considerable attention in the last decade^15^, ^20^, ^30^, ^50^, ^51^, ^52^, ^53^, ^54^ and might be used in the future, apart for FLASH RT, also to target very small and deep tumor volumes through magnetic focusing,^16^, ^51^, ^55^ or to be combined with spatially fractionated RT, as grid or minibeam RT.^56^, ^57^, ^58^

Our study presents some limits affecting the accuracy of the recombination measurement, which we incorporated in the results through an evaluation of the possible sources of uncertainties. We have followed the IAEA TRS‐398 protocol methodology to assess our dosimetry because it is a widely used reference protocol for clinical electron beam dosimetry. However, it can be noted that this protocol was not fully applicable in our experimental conditions. Indeed, we chose to use the solid water RW3 material as reference material instead of water for convenience in UHDR measurements, the material choice not modifying the result of ICE. In addition, the RNC has a too small sensitive volume to fit the protocol's criteria, but this chamber was chosen on purpose to take advantage of its very small sensitive volume to reduce the recombination effect in UHDR conditions. Moreover, the use of an experimental facility to reach UHDR irradiation did not allowed to determine the correction factors with the same precision than achievable in conventional conditions, in particular to decorrelate the collection efficacy from the polarity uncertainties. Therefore, we followed the methodology of the TRS‐398, within the limits of these nonstandard experimental conditions. Besides, the choice of using a polarization factor of 1 in conventional mode on the clinical Synergy Elekta LINAC is justified by the very small impact expected using a large field of 20 × 20 cm^2^, as a maximum polarization correction of 0.2% was reported by Looe et al.^35^ using the RNC with such field sizes.

Finally, as our method to determine the ICE is based on a measurement coupling two different detector types, the sources of uncertainties are potentially cumulated. In particular, the response of the RNC and of the radiochromic films might not change in the same way with the machine fluctuations or beam parameters (pulse duration, grid tension, and beam size at different SSD), in particular if those parameters affect the energy spectrum. Petersson et al.^32^ proposed another method to evaluate the ICE (cf. Figure 5) exclusively based on the ion chamber response. Their method assumes that the impact of recombination at the lowest GT at all SSD used is negligible, and that the ratio between the charge collected by the chamber at two different GT does not change with SSD unless recombination occurs. Applying the method described by Petersson et al. to our measurement series on which the previous assumption was valid leads to ICE values that are coherent with the first method, with recombination starting to appear at DPP above 0.1 Gy/pulse, but giving higher ICE values for higher DPP than those obtained and presented in the current study. Hence, the conclusions would be even more favorable for the RNC in terms of reduced recombination for FLASH RT.

## 5 CONCLUSIONS

This study was focused on determining the recombination correction factor of the IBA RNC in very high dose‐rate irradiations. The results confirmed that the small sensitive volume of the RNC ensures higher ICE compared with larger chambers. Notably, we measured an ICE of the RNC above 55% at extreme dose per pulses of 10 Gy and we determined a recombination model for the RNC in the range of 0.01–30 Gy/pulse. The measurements also demonstrated a higher sensitivity to the pulse duration of the RNC compared with larger chambers for microsecond pulses. Further studies will be dedicated to a deeper investigation of the pulse duration impact on the ionization chamber response, in particular to extrapolate these results for short pulse durations down to femtoseconds. To conclude, the reduced sensitivity to recombination effects and the compact dimensions of the RNC makes this chamber an appealing tool for FLASH RT as well as for stereotactic or spatially fractionated RT applications involving small fields with potential high dose rates.

## CONFLICT OF INTEREST

All the authors declare that they have no conflict of interest in relation to the research in the submitted manuscript.
